# Implementation of a national guideline for analgesia and sedation: how often can a RASS of 0 to -2 be achieved?

**DOI:** 10.1186/cc10932

**Published:** 2012-03-20

**Authors:** R Riessen, P Tränkle, R Pech, G Blumenstock, M Haap

**Affiliations:** 1 University Hospital Tübingen, Germany

## Introduction

Based on a new national guideline we implemented in our medical ICU an interdisciplinary algorithm for the management of analgosedation, in which nurses had to adjust the dose of the analgesics and sedatives based on sedation goals given by the physicians. Within this project we investigated in what portion of mechanically ventilated patients a sedation level of Richmond Agitation and Sedation Scale (RASS) of 0 to -2, which is generally recommended by the guideline, can be achieved. We also asked the nurses for an explanation when this goal was not reached.

## Methods

After an educational program the level of sedation was measured 364 times in 37 mechanically ventilated patients at different time points by an independent observer. In all cases in which the RASS was outside the desired level of 0 to -2, the nurse in charge was asked to fill out a structured as well as open questionnaire, in which the reasons for this deviation could be stated.

## Results

The independent observer documented only in 13% (47/364) of all measurements a RASS of 0 to -2. We analyzed 295 questionnaires, in which 368 reasons for a deviation from a RASS of 0 to -2 were stated (multiple answers were possible). In 113 questionnaires (38%) the nurses mentioned that a short-term increase in sedation depth was required for nursing procedures or medical interventions. In 89 questionnaires (30%) a RASS of 0 to -2 was considered reasonable but could not be achieved at the time of measurement with the current medication (*n *= 32) or the consciousness was impaired by CNS diseases (*n *= 52). In 100 questionnaires (34%) a RASS of 0 to -2 was not considered reasonable. The following reasons were stated: disease with coma (*n *= 25), controlled ventilation (*n *= 32), distressed patient (*n *= 12), increased intracranial pressure (*n *= 7), status epilepticus (*n *= 7), hypothermia (*n *= 4), dying patient (*n *= 4), delirium/(auto) aggression (*n *= 4). Other reasons were mentioned in 66 questionnaires (22%), most commonly a physician order for a deeper sedation (*n *= 19) or a missing sedation goal (*n *= 14).

## Conclusion

In mechanically ventilated patients of a medical ICU including also patients with neurologic diseases, a sedation goal of RASS 0 to -2, as recommended by a current guideline, could only be achieved in a minority of patients despite intensive instructions and a motivated team. In most cases the nurses were able to provide reasonable medical explanations for a deeper sedation or an otherwise impaired consciousness.

